# Omega-3 fatty acid-rich fish oil supplementation prevents rosiglitazone-induced osteopenia in aging C57BL/6 mice and in vitro studies

**DOI:** 10.1038/s41598-021-89827-8

**Published:** 2021-05-14

**Authors:** Chiara Cugno, Dhanya Kizhakayil, Rita Calzone, Shaikh Mizanoor Rahman, Ganesh V. Halade, Md M. Rahman

**Affiliations:** 1 Advanced Cell Therapy Core, Sidra Medicine, Doha, Qatar; 2 Natural & Medical Sciences Research Center, University of Nizwa, Nizwa, Sultanate of Oman; 3 Division of Cardiovascular Sciences, The University of South Florida Health, Tampa, FL USA; 4 Department of Biological and Environmental Sciences, Qatar University, Doha, Qatar

**Keywords:** Drug safety, Mesenchymal stem cells, Endocrine system and metabolic diseases

## Abstract

Rosiglitazone is an effective insulin-sensitizer, however associated with bone loss mainly due to increased bone resorption and bone marrow adiposity. We investigated the effect of the co-administration of fish oil rich in omega-3 fatty acids (FAs) on rosiglitazone-induced bone loss in C57BL/6 mice and the mechanisms underlying potential preventive effect. Mice fed the iso-caloric diet supplemented with fish oil exhibited significantly higher levels of bone density in different regions compared to the other groups. In the same cohort of mice, reduced activity of COX-2, enhanced activity of alkaline phosphatase, lower levels of cathepsin k, PPAR-γ, and pro-inflammatory cytokines, and a higher level of anti-inflammatory cytokines were observed. Moreover, fish oil restored rosiglitazone-induced down-regulation of osteoblast differentiation and up-regulation of adipocyte differentiation in C3H10T1/2 cells and inhibited the up-regulation of osteoclast differentiation of RANKL-treated RAW264.7 cells. We finally tested our hypothesis on human Mesenchymal Stromal Cells differentiated to osteocytes and adipocytes confirming the beneficial effect of docosahexaenoic acid (DHA) omega-3 FA during treatment with rosiglitazone, through the down-regulation of adipogenic genes, such as adipsin and FABP4 along the PPARγ/FABP4 axis, and reducing the capability of osteocytes to switch toward adipogenesis. Fish oil may prevent rosiglitazone-induced bone loss by inhibiting inflammation, osteoclastogenesis, and adipogenesis and by enhancing osteogenesis in the bone microenvironment.

## Introduction

Thiazolidinediones (TZDs, also known as glitazones) are a class of antidiabetic agents that act as insulin sensitizers. Rosiglitazone (RSG) and pioglitazone are the most commonly used anti-diabetic drugs of this class and they work by binding to the peroxisome proliferator-activated receptors (PPAR)-γ in fat cells and making the cells more responsive to insulin^1^.

TZDs are recommended in the algorithm of the American College of Endocrinologists for the treatment of Type 2 Diabetes (T2D), as per the latest consensus statement in January 2020^2^. They are the only antihyperglycemic agents able to reduce insulin resistance, in addition to potent A1C-lowering properties associated with low risk of hypoglycemia and durable glycemic effects^2–4^. Pioglitazone may also confer cardiovascular diseases (CVD) benefits^3,5,6^, while RSG has a neutral effect on CVD risk^7,8^. However, the use of TZDs has been limited by the onset of side effects, such as weight gain, increased bone fracture risk in postmenopausal females and elderly males, and an elevated risk for chronic edema or heart failure^2,9–12^.

RSG (Avandia^®^, GlaxoSmithKline) is a potent TZD insulin sensitizer that decreases hyperglycemia by reducing insulin resistance in patients with T2D^4^. It is used as a stand-alone drug or in combination with metformin or glimepiride. Despite RSG’s effectiveness at decreasing blood sugar in T2D, its use decreased dramatically as studies showed apparent associations with increased risks of heart attacks and death^13^. Although extensive controversies raised by the detection of side effects, especially increased risk of heart failure^9^, the Food and Drug Administration (FDA) decided to keep the drug available in the US; from November 2011 until November 2013, Avandia^®^ was sold only with a prescription from a certified doctor. In November 2013, the FDA lifted its earlier restrictions on RSG after reviewing the results of the 2009 RECORD (Rosiglitazone Evaluated for Cardiac Outcomes and Regulation of glycemia in Diabetes) clinical trial: a 6-year, open-label randomized control study that did not show any significant effect of RSG on myocardial infarction, being also the risk of overall cardiovascular morbidity or mortality comparable to other standard glucose-lowering drugs^7^.

Despite the controversies on the risk profile side effects, RSG remains a drug of choice for many T2D patients because of its unique insulin-sensitizing capacity^2^. Side effects variably associated with RSG include increased bone loss and adiposity, risk of myocardial infarction and other CVD^14,15^, and decreased serum bone-specific alkaline phosphatase (ALP) in older diabetic patients^16^. The long-term follow-up of the RECORD 2009 study showed that, consistently with the main study, RSG is associated with an increased risk of peripheral bone fracture in women, and probably in men, however without an increase in potentially high-morbidity (hip, pelvis, femur, and spine) fractures^17^.

A review of fracture risk associated with TZDs therapy in preclinical studies demonstrated that activation of PPARγ inhibits bone formation primarily by diverting Mesenchymal Stromal Cells (MSCs) to the adipocytic rather than to the osteogenic lineage and that TZDs may also increase bone resorption by stimulating osteoclasts^18^. Increased bone resorption due to up-regulation in osteoclastogenesis and decresead bone formation due to down-regulation of osteoblastogeneis are the key to bone loss associated with inflammatory diseases^19^.

Considered that the safety of this powerful therapeutic agent is still under strict scrutiny, there is an imperative need to develop preventive strategies to reduce RSG-mediated bone loss in T2D patients. Both T2D and osteopenia are considered to be inflammatory disease^20,21^ and fish oil (FO) rich in omega-3 fatty acids (FAs) are natural anti-inflammatory compounds^22–24^. FO has been shown to reduce the incidence of a wide range of degenerative disorders and medical problems, including CVD and sudden death in epidemiological and clinical trials^25,26^. In 2004, FO was recognized and approved by the FDA for the treatment of hyperlipidemia (LOVAZA™ Reliant Pharmaceuticals, now, GSK Inc, USA). Potential beneficial actions of FO on the cardiovascular system^25,26^ and numerous non-cardiac conditions, including inflammatory and arthritic disorders^27^, neurological/neuropsychiatric disorders, ophthalmic disorders, gynecological problems, cancer^28^, autoimmune disease, and osteoporosis^24^ have been investigated^29^.

Earlier, we have demonstrated that dietary supplementation with FO protects against ovariectomy-induced bone loss at the femur and lumbar vertebrae in mice^30^. We hypothesize that this protection is due to omega-3 polyunsaturated fatty acids (omega-3 PUFAs), especially eicosapentaenoic acid (EPA; C20:5n-3) and docosahexaenoic acid (DHA; C22:6n-3), richly found in FO, which, as PPARs regulators, are relevant in the prevention and treatment of pathologies related to aging^31^. In humans, elevated concentration of omega-3 FAs was positively correlated with Bone Mineral Density (BMD)^32^. Additionally, in-vitro studies using the preosteoblastic cell line MC3T3-E1 indicated that the production of the bone-formation markers, ALP and osteocalcin, were higher after 48 h of treatment with EPA, compared with arachidonic acid C20: 4n-6 (AA)^33^. Furthermore, we have confirmed that DHA is even more potent than EPA in inhibiting the receptor activator of nuclear factor kappa-Β ligand (RANKL)-stimulated osteoclast differentiation, activation, and function in the RAW 264.7 cell line^34^. These in-vitro and in-vivo animals and human studies have suggested that FO may attenuate inflammation and reduce RSG-mediated postmenopausal bone loss. However, whether FO can alleviate RSG-mediated increase in bone loss has not been previously explored.

In the current study, we have conducted three separate sets of experiments in order to evaluate efficacy and elucidate potential mechanisms of FO in the prevention of RSG-mediated bone loss:

- In vitro, we explored possible mechanisms by which the FO components, EPA and DHA, might reduce RSG-associated bone resorption. In particular, we evaluated the RANKL-stimulated osteoclast differentiation in RAW264.7 cells and the osteoclastic activity on sperm whale dentin slice, induced by parathyroid hormone-related protein (PTHrP) in young mouse bone marrow (BM) cells. Osteogenesis and adipogenesis induced by bone morphogenic protein-2 (BMP-2) in C3H10T1/2 cells, and osteogenesis stimulated by ascorbic acid and β-glycerophosphate in mouse BM stromal cells in-vitro were also examined.
- In vivo, we investigated the protective effects of FO against RSG-mediated bone loss in aging C57Bl/6 mice, a strain susceptible to obesity, hyperglycemia, and insulin resistance when fed corn oil (CO)-based diet^35^.
- Finally, as an assay of human readout, we tested the effect of DHA on human adipose tissue-derived MSCs (AD-MSCs) during differentiation towards osteocytes and adipocytes in vitro, showing how gene expression is modulated.

## Results

### In-vitro experiments

#### Omega-3 FAs attenuate RSG-mediated stimulation of adipogenesis and reduction of osteoblast differentiation in C3H10T1/2 cells

**Figure 1:**
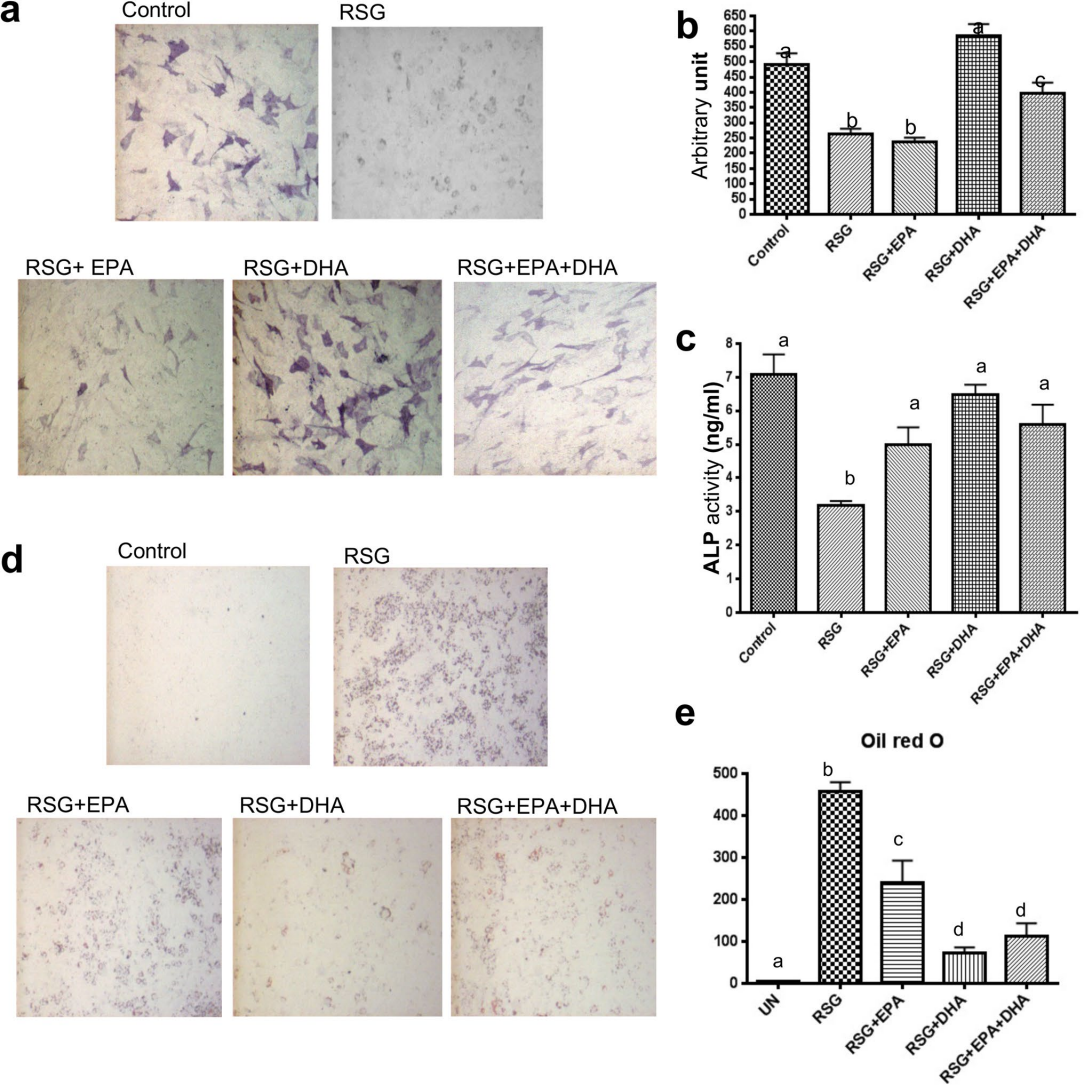
Effect of RSG with or without omega-3 FAs on osteoblastogenesis and adipogenesis in C3H10T1/2 cells. C3H10T1/2 cells were plated in a 48-well plate and cultured in α-MEM medium with 10% FBS supplemented with recombinant human (rh) BMP-2 (Biovision, USA) and cultured for 7 days in the presence of RSG 1 µM with or without EPA 10 µM and/or DHA 10 µM. Representative photographs (20×) of C3H10T1/2 cells stained for alkaline phosphatase (elongated cells) (**a**). Optical density measured by Metaview Image Analysis (Olympus Inc. USA) in RSG treated cells with and without FO (EPA and DHA) in the presence of BMP-2 (**b**). Cells were lysed with lysis buffer and quantified for protein content. Alkaline phosphatase (ALP) activity was measured by using SensoLyte pNPP ALP Assay by mixing cell lysates with pNPP reaction mixture and incubated for 30 min. ALP activity in each sample was determined by measuring OD405 and compared to an ALP standard curve (**c**). Cells were then stained with Oil red O using fast red AL salt as a substrate. Representative photographs (20x) of C3H10T1/2 cells stained for Oil red O (round cells) (**d**). Optical density measured by Metaview Image Analysis (Olympus Inc. USA) in RSG-treated cells with and without FO (EPA and DHA) supplementation (**e**). Each bar represents the mean ± SEM of 6 wells per group. Value with different superscripts is significantly different at p < 0.05 by Newman–Keuls’ one-way ANOVA with multiple comparison tests. *ALP* Alkaline phosphatase, *RSG* rosiglitazone, *EPA* eicosapentaenoic acid, *DHA* docosahexaenoic acid, *MEM* minimum essential medium, *BMP-2* bone morphogenetic protein 2.

C3H10T1/2, a cell line established from an early mouse embryo with a high degree of sensitivity to post-confluence inhibition^36^, has been extensively used as an in-vitro model to examine mesenchymal differentiation into various phenotypic lineages by different inductive mediators^37^. It has been demonstrated in multiple studies that, on bone morphogenetic protein (BMP)-2 stimulation, C3H10T1/2 cells undergo differentiation to an osteogenic phenotype^38,39^. ALP is an important marker for osteoblast activity during early osteoblast differentiation. In this study, we exposed confluent C3H10T1/2 cells to recombinant human (rh)BMP-2 in the presence of RSG with or without omega-3 FAs and determined the effect on osteoblast differentiation by ALP activity. Our results demonstrated that RSG significantly reduced the BMP-2-stimulated osteoblast differentiation and omega-3 FAs co-administration restored it (Fig. 1). We also determined the effect of RSG with or without omega-3 FAs on adipogenesis in C3H10T1/2 cells stimulated with rhBMP-2. Our results showed that RSG induces the adipocyte differentiation in C3H10T1/2 cells and that BMP-2-induced adipogenesis is significantly higher in RSG-treated cells (Fig. 1). However, omega-3 FAs co-administration could attenuate this effect (Fig. 1). Interestingly, the effect of DHA was stronger than EPA in reducing adipogenesis, indicating that omega-3 FA-rich FO has the potential to be an effective adjuvant dietary supplement with the RSG for T2D to attenuate RSG-mediated stimulation of adipogenesis and reduction of osteogenesis.

#### Effect of RSG with or without omega-3 FA on osteogenesis in BM stromal cells

We further tested the effect of RSG with or without omega-3 FA on osteogenesis in ascorbic acid- and β-glycerophosphate-treated BM stromal cells by measuring the levels of matrix calcification through alizarin red staining and osteoblastic activity by ALP activity. Our results showed that RSG significantly reduced the matrix calcification in BM stromal cells and omega-3 FAs co-administration restored it to as of control. Similarly, RSG significantly reduced the ALP activity and omega-3 FAs co-administration reinstated it (Fig. S1).

#### Effect of RSG with or without omega-3 FAs on osteoclastogenesis in RAW 264.7 cells

**Figure 2:**
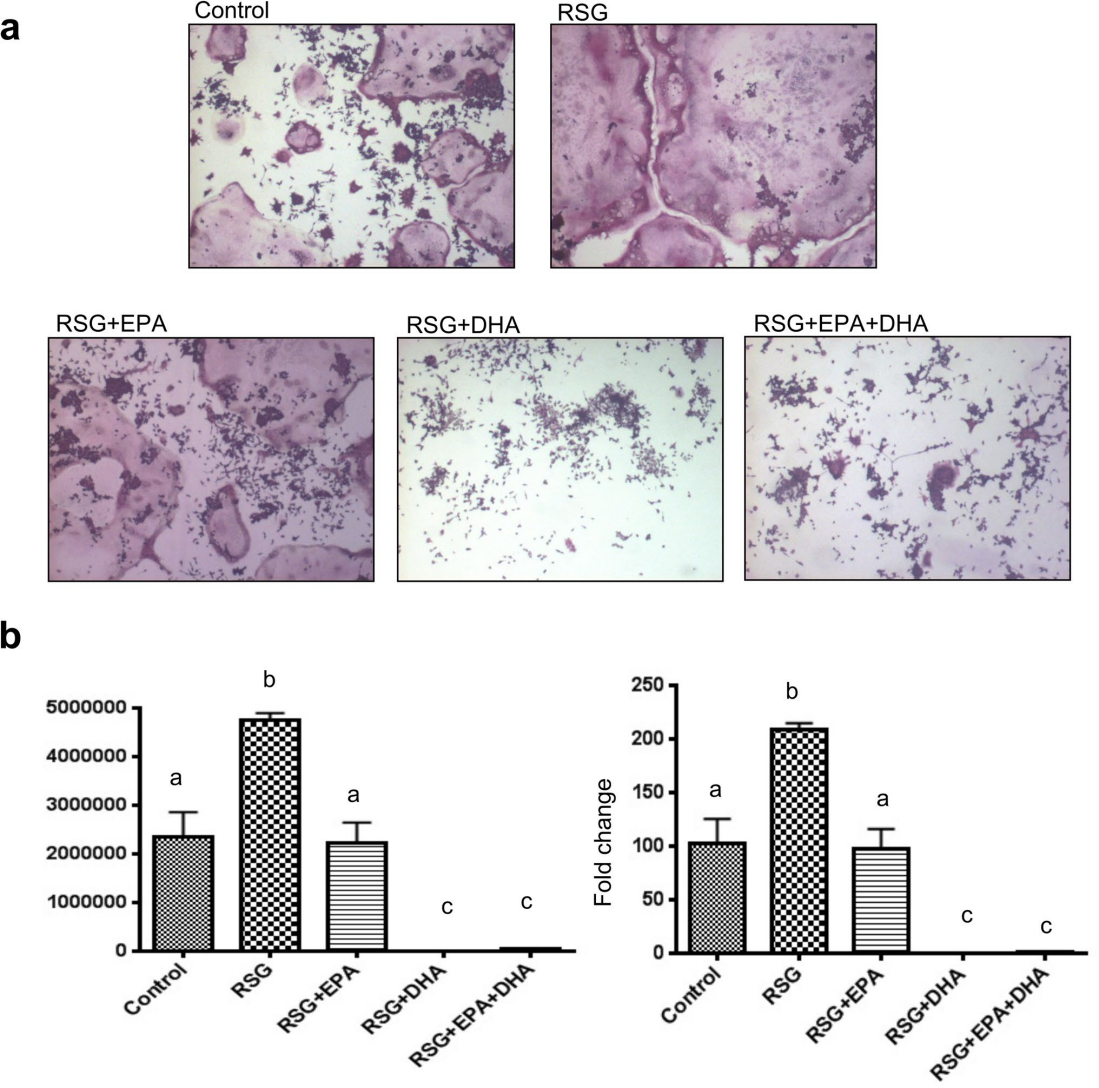
Effect of RSG with or without omega-3 FAs on osteoclastogenesis in RAW 264.7 cells. RAW 264.7 cells were cultured with RANKL 50 ng/mL in the presence of RSG 1 µM with or without EPA 10 µM and/or DHA 10 µM for 5 days. At the end of the cultures, cells were fixed and stained for tartrate-resistant acid phosphatase (TRAP). The number of TRAP-positive multinucleated cells (MNCs) (more than 3 nuclei) was counted as osteoclast-like cells in each well. Representative photographs (20×) of TRAP-positive MNCs (**a**). Optical Density (OD) intensity was determined by histomorphometry using the Metaview Image Analysis System software for the TRAP-positive area (**b**). Each bar represents the mean ± SEM of two independent quadruplicate cultures. Value with different superscripts is significantly different at p < 0.05 by Newman–Keuls one-way ANOVA with multiple comparison test. *RSG* rosiglitazone, *EPA* Eicosapentaenoic acid, *DHA* Docosahexaenoic acid, *RANKL* Receptor activator of nuclear factor kappa-Β ligand.

Bone density depends on the delicate balance of three cell lineages: osteoblasts, adipocytes, and osteoclasts. Therefore, we determined the effect of RSG with or without omega-3 FAs on osteoclasts formation in RAW 264.7 cells stimulated with RANKL, by tartrate-resistant acid phosphatase (TRAP) staining. Our results showed that RSG significantly enhanced the osteoclast differentiation in RAW 264.7 cells, and omega-3 FAs co-administration restored it to as of control (Fig. 2). Interestingly, the effect of DHA was stronger than EPA in reducing osteoclastogenesis even significantly less than control (Fig. 2). This data supports our previously published study that DHA is a more potent inhibitor of osteoclast differentiation than EPA by suppressing cell fusion^34^. Whereas, in the present study, we found that RSG promoted cell fusion to make giant osteoclasts (Fig. 2).

#### Effect of RSG with or without omega-3 FAs on osteoclastic activity in BM cells

We further tested the effect of RSG with or without omega-3 FAs on osteoclastic activity on dentin slice using BM cells stimulated with PTHrP by measuring TRAP activity. Our results showed that omega-3 FAs co-administration attenuated RSG-mediated activation of osteoclastic activity of BM cells on dentin slice (Fig. S2). As seen in other experiments, DHA had a better anti-osteoclastogenic effect when combined with RSG than that with EPA (Fig. S2). These data indicate that omega-3 FAs rich FO has the potential to be an effective adjuvant dietary supplement, able to attenuate RSG-mediated stimulation of osteoclastogenesis, and thus to reduce bone resorption.

### In vivo experiments

#### Bodyweight, food intake, and serum metabolites

All experimental groups were provided with an equivalent amount of daily calories using the Lab Chow (LC) standard rodent diet as the control; the different dietary treatments did not affect food intake in any group. However, bodyweight gain was ~ 33% higher in CO-fed mice compared to a ~ 3% increase in the LC-fed mice. This is consistent with the well-characterized obesity, insulin resistance, and T2D seen previously in these mice when fed a fat-supplemented diet^35^. The RSG + CO, FO, and RSG + FO mice showed a ~ 10% decrease in body weight gain compared to that of the CO-fed mice, even though there was no difference in food intake between these groups. Insulin resistance, which is strongly associated with obesity, is thought to be also caused by metabolic messengers, such as free FAs, produced by adipose tissue that inhibit insulin action on muscle^40^. Furthermore, non-esterified fatty acid (NEFA) levels were significantly decreased in RSG + CO and RSG + FO compared to CO-fed mice. Increased fasting insulin and decreased glucose values in RSG + CO and RSG + FO fed mice compared to CO fed mice were observed (Table 1). Interestingly, the addition of RSG with FO did not alter the pharmacological activity in controlling hyperglycemia. This finding confirms the compatibility of RSG with FO and indicates the absence of a diet-drug interaction.

**Table 1:** Body weight and serum metabolites in C57Bl/6J aging mice after 5 months’ dietary supplementation with CO, FO and/or RSG, respectively, compared with LC controls.

Parameters	I CO	II FO	III RSG + CO	IV RSG + FO	V LC
**Body weights (n = 15)**					
Body weight (g)					
Pre	23.7 ± 0.94	23.2 ± 0.50	23.2 ± 1.12	23.3 ± 0.72	23.5 ± 0.75
Post	31.7 ± 1.2(33)^a^	28.5 ± 1.21(23)^b^	28.9 ± 0.87(24)^b^	28.7 ± 0.8(24)^b^	24.2 ± 0.81(3)^b^
**Serum metabolites (n = 10)**					
Glucose (mg/dl)	194.1 ± 7.2	157.2 ± 4.2	129.6 ± 4.3*	121.0 ± 6.3*	80.12 ± 4.2
Insulin (ng/mL)	0.32 ± 0.04	0.42 ± 0.05	0.68 ± 0.1*	0.72 ± 0.1*	0.10 ± 0.04
NEFA (mmol/L)	1.27 ± 0.03	0.90 ± 0.02	0.87 ± 0.05*	0.76 ± 0.05*	1.06 ± 0.12

Values in parentheses are the differences between the pre- and post-supplementation values.

Values with different letters, and sign indicate that values are significantly different by ANOVA, followed by Newman–Keuls multiple comparison post hoc test (p < 0.05).

*p <  0.05 compared to CO-fed mice control.

#### Prevention of RSG-mediated BMD loss by FO in C57BL/6 aging mice

**Figure 3:**
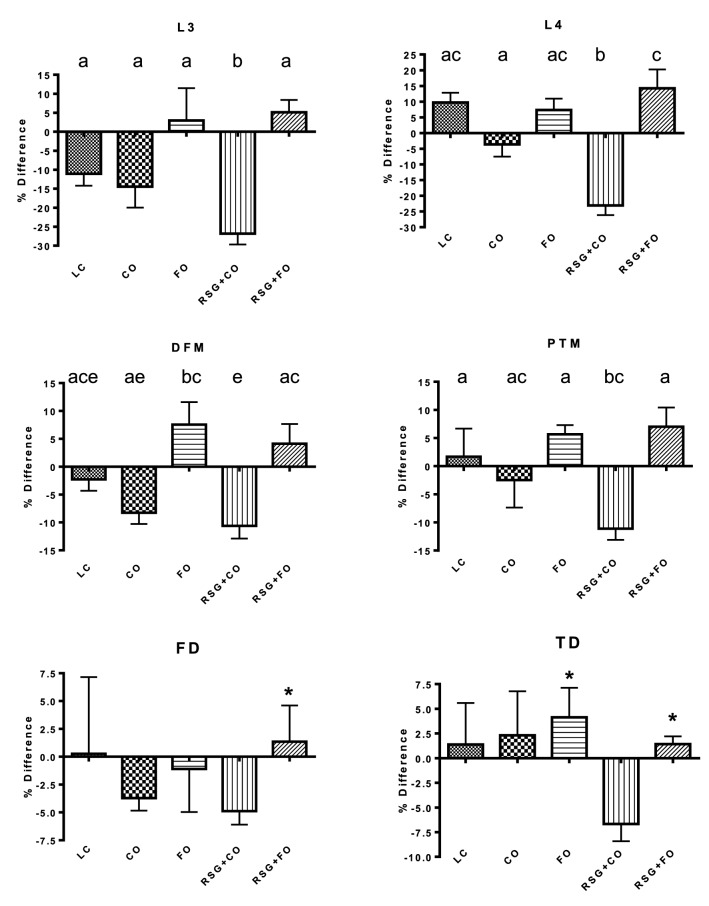
Effect of RSG with or without FO on bone mineral density (BMD) of aging C57BL/6 mice. Thirteen months old C57Bl/6 mice (n = 10) were fed with CO, RSG + CO, FO, RSG + FO, and LC for 5 months. Region-specific BMD was measured by dual-energy x-ray absorptiometry (DEXA) for lumbar vertebra 3 (L3), lumbar vertebra 4 (L4), distal femoral metaphysis (DFM), proximal tibial metaphysis (PTM), femoral diaphysis (FD), and tibial diaphysis (TD). A DEXA scan was performed at 13 months of age just before starting the experimental diets to determine the baseline BMD value. Thirteen-month-old C57Bl/6 mice were fed experimental diets for 5 months and then another DEXA scan was performed to determine the endpoint BMD value. The percent difference in BMD value was analyzed from the endpoint value to the baseline value. Each value represents the mean ± SEM. n = 8–10 mice per group. Values with different letters are significantly different by one-way ANOVA followed by Newman-Keuls multiple comparison post hoc tests (p < 0.05). *CO* corn oil, *RSG* rosiglitazone, *FO* fish oil, *LC* lab chow.

BMD was measured at 13 months of age (baseline) and after 5 months of experimental diets (final value). Data were expressed as percent difference in BMD and analyzed from the endpoint value to the baseline value. Following 5 months of dietary supplementation with FO, aging RSG-treated female C57Bl/6 mice displayed increases of 5–7% in BMD of the spine, distal femoral metaphysis (DFM), and proximal tibial metaphysis (PTM). These increases were in striking contrast (10–28% *vs* 5–10%) to the decline in BMD displayed by the RSG + CO mice, even greater than those observed in CO and LC mice. RSG + CO-fed mice showed an accelerated loss in BMD compared to control CO and LC-fed mice (Fig. 3). A notable reduction of BMD in all regions of bone was observed in RSG-treated mice (Fig. 3). The RSG-treated mice exhibited the lowest BMD in the PTM, femoral diaphysis, tibial diaphysis, and the lumbar regions of the spine as compared to that of all the other groups. Interestingly, this reduction of BMD levels in all bone regions was restored when RSG was co-administered with FO (Fig. 3). These data support our in vitro findings that omega-3 FAs could attenuate the negative regulation of RSG on osteogenesis and adipogenesis, thus preventing RSG-mediated bone loss in animal models.

### Ex-vivo

#### Effect of RSG with or without FO on bone markers of aging C57BL/6 mice

**Figure 4:**
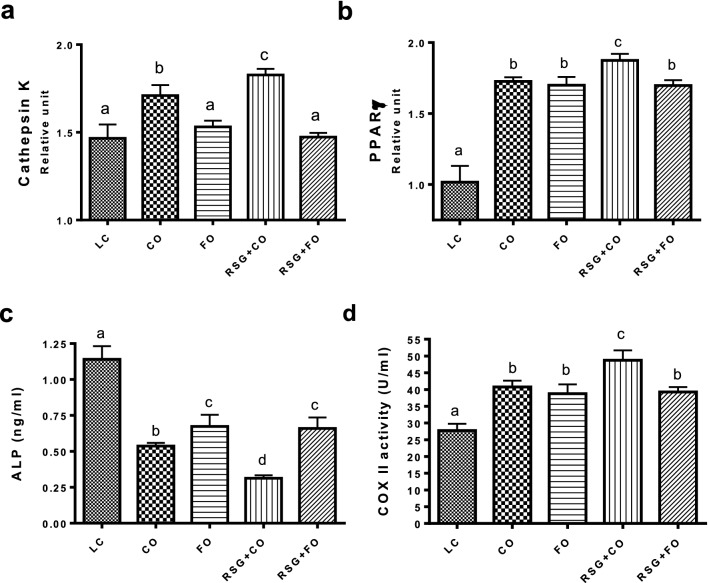
Effect of RSG with or without FO on bone markers of aging C57BL/6 mice. At sacrifice whole bone marrow (BM) cells were collected from aging C57Bl/6 mice (n = 6) of each experimental diet group: CO, RSG + CO, FO, RSG + FO, or a standard lab chow control diet (LC) fed for 5 months. BM cells were treated with LPS for 24 h and RNA was prepared from cell pellets; gene expression of cathepsin k (**a**) and PPARγ (**b**) was determined by real-time RT-PCR. Cell lysates of whole BM cells from different experimental groups treated with LPS were also used to determine Alkaline phosphatase activity (**c**) and COX-II activity (**d**). Values with different letters are significantly different by one-way ANOVA followed by Newman-Keuls multiple comparison post hoc tests (p < 0.05). *CO* corn oil, *RSG* rosiglitazone, *FO* fish oil, *LC* lab chow, *PPARγ* peroxisome proliferator-activated receptor-gamma, *COX-II* Cyclooxygenase-2.

After 5 months of experimental diets, BM cells were collected and stimulated with lipopolysaccharide **(**LPS). Quantitative real-time RT-PCR demonstrated that LPS-stimulated mRNA expression of both cathepsin k (marker of bone resorption) and PPARγ (marker of fatty bone formation) was significantly increased in BM cells collected from RSG-treated animals and re-established in BM cells collected from the RSG + FO group (Fig. 4a,b). FO supplementation, either alone or in combination with RSG, decreased age-associated and RSG-mediated osteoclastogenic bone resorption, thus improving BMD (Fig. 4a). Furthermore, PPARγ was significantly increased in RSG mice compared with RSG + FO mice (Fig. 4b). Cell lysates of whole BM cells from different experimental groups treated with LPS were also used to determine ALP (Fig. 4c) and cyclooxygenases (COX)-II activity (Fig. 4d). We noted that ALP activity was significantly decreased in RSG + CO mice, compared with CO and LC mice (Fig. 4c) and that FO supplementation among RSG-treated mice significantly blunted the RSG-related reductions in ALP activity in cultured BM. Levels of COX-II, which is known to stimulate osteoclastogenesis and bone resorption^41^, were increased in the BM of CO and RSG + CO-fed mice, whereas FO was shown to down-regulate COX-II expression, as a possible mechanism by which osteoclastogenesis and RSG-mediated bone resorption might be attenuated. Interestingly, we found a significant reduction of COX-II activity in LPS-treated BM cells from FO supplemented RSG mice, compared with CO and RSG + CO mice (Fig. 4D).

#### Effect of RSG with or without FO on inflammatory markers of aging C57BL/6 mice

**Figure 5:**
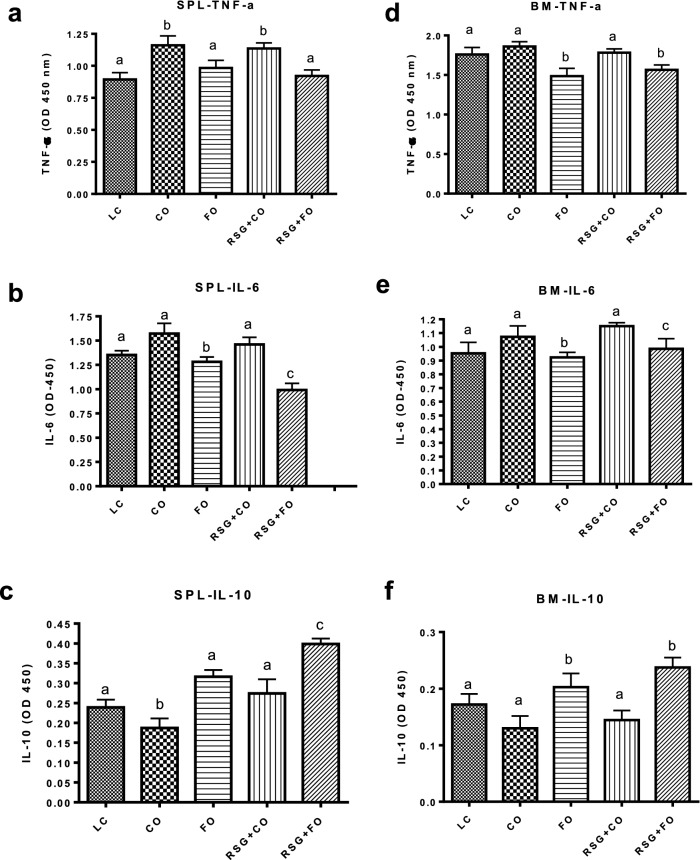
Effect of RSG with or without FO on inflammatory markers of aging C57BL/6 mice. Splenocytes and whole bone marrow (BM) cells were isolated from aging C57BL/6 mice on experimental diets: CO, RSG + CO, FO, RSG + FO, and LC for 5 months. Splenocytes (**a–c**) and BM cells (**d–f**) were cultured separately and treated with LPS for 24 h and culture supernatants were collected and analyzed for pro-inflammatory TNF-α, and IL-6 and anti-inflammatory IL-10 cytokines. n = 5 mice per group. Values with different letters are significantly different by one-way ANOVA followed by Newman-Keuls multiple comparison post hoc tests (p < 0.05). *CO* corn oil, *RSG* rosiglitazone, *FO* fish oil, *LC* lab chow, *LPS* Lipopolysaccharide, *TNF-α* Tumor necrosis factor, *IL-6* Interleukin-6, *IL-10* Interleukin-10.

Splenocytes and whole BM cells isolated from aging C57BL/6 mice on experimental diets were stimulated with LPS and analyzed for pro-inflammatory and pro-osteoclastogenic TNF-α and IL-6, and anti-inflammatory and anti-osteoclastogenic IL-10 cytokines. Interestingly, we found a significant decrease in IL-6 and TNF-α, and elevated IL-10 production by LPS-treated splenocytes (Fig. 5a–c) and BM cells (Fig. 5d–f) from FO and RSG + FO mice, compared with those from CO and RSG + CO mice. These results indicate that the reduction of pro-inflammatory cytokines by BM and splenocytes may indirectly prevent RSG-mediated bone loss by inhibiting bone-resorbing osteoclastogenesis in aging mice. Accordingly, increased levels of IL-10 production by splenocytes and BM from FO and RSG + FO mice, compared with those of RSG + CO mice, may have prevented RSG-mediated bone loss by suppressing bone resorption.

### Human readout in vitro

AD-MSCs consistently (more than 50% in all conditions) differentiated in osteocytes; the differentiation rate for adipocytes was lower (5–9%), as expected for the assay. The ratios between differentiated, stained regions and total areas were calculated through the ImageJ software and no significant changes were identified between RSG and RSG + DHA, both in the adipo- and osteo-differentiation conditions (Fig. S3).

**Figure 6:**
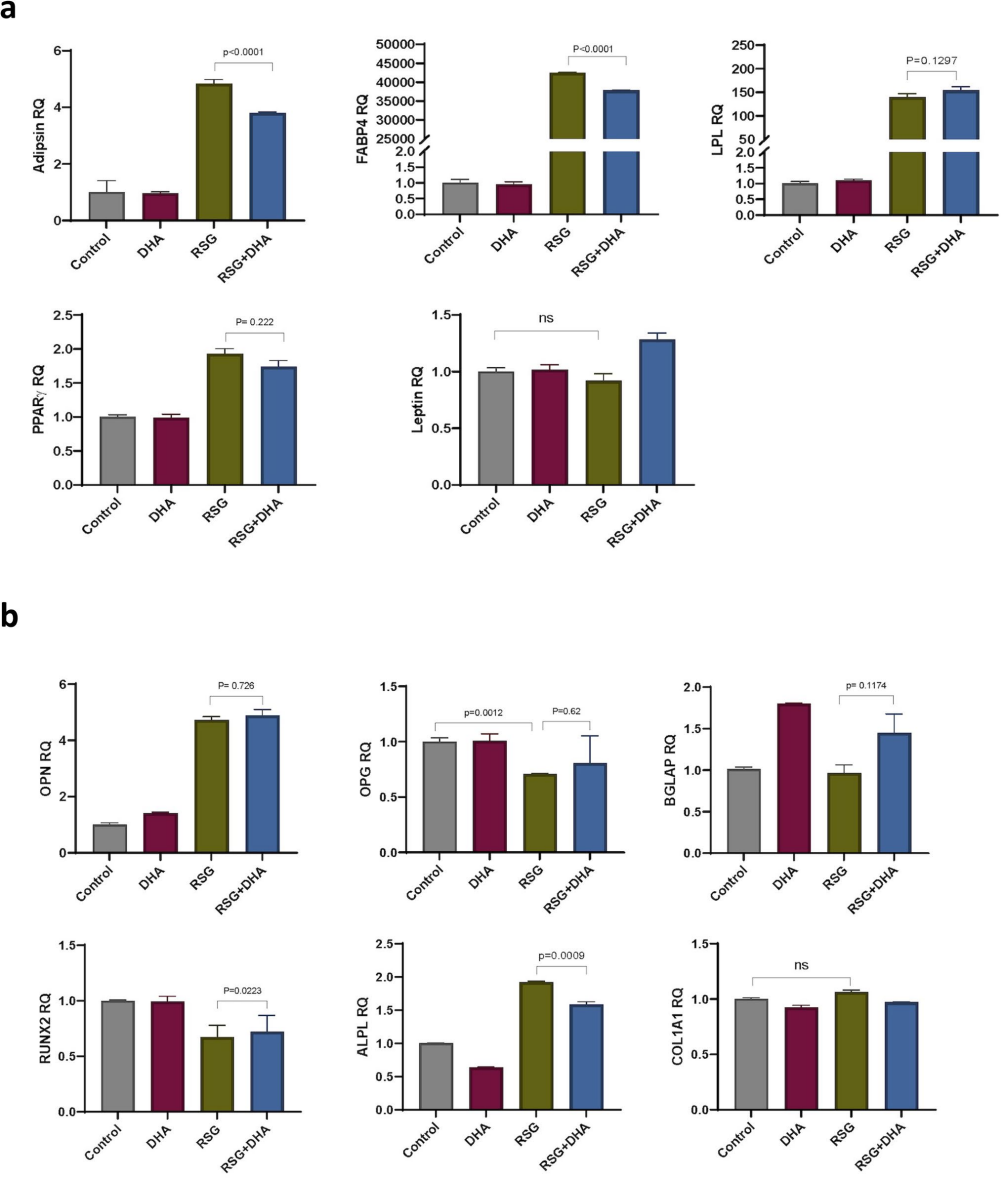
Effect of RSG with or without FO on gene-expression of osteo-differentiated Mesenchymal Stromal Cells (MSCs). Gene expression of adipogenic (**a**) and osteogenic (**b**) markers in MSC differentiated in osteogenic medium with the presence or absence of RSG, DHA, and combination of RSG and DHA. Relative quantification of gene expression for Adipsin, FABP4, LPL, PPARγ, Leptin, OPN, OPG, BGLAP, RUNX2, ALPL, and COL1A1. Each bar represents the RQ mean ± 95% CI from two donors in triplicates. Value with different superscripts is significantly different at p < 0.05 by one-way ANOVA test followed by Dunnett’s multiple comparisons tests with RSG group. *ns* p-value non-significant, *RSG* rosiglitazone, *EPA* eicosapentaenoic acid, *DHA*, docosahexaenoic acid.

The analyses of the broad gene panel covering adipogenesis and early and late osteogenesis showed that the addition of DHA reduces the relative expression of adipsin (p < 0.0001), fatty acid-binding protein (FABP)-4 (p < 0.0001), Runt-related transcription factor (RUNX)-2 (p = 0.0223) and ALP (p = 0.0009), with a not statistically significant trend for PPARγ (p = 0.222) in the osteogenic assay (Fig. 6). Adipsin, or complement factor D, is an adipocyte-specific gene. It functions as an adipokine, a cell-signaling protein secreted by adipocytes, which regulates insulin secretion in mice, and promotes lipid accumulation and adipocyte differentiation. FABP4 encodes the fatty acid-binding protein found in adipocytes.

In the adipogenic setting, the increase of FABP4 (p = 0.0017) and OPG (p = 0.0051) reached statistical significance (Fig. S4).

## Discussion

Evidence suggests that chronic inflammatory diseases of almost any cause are associated with the bone loss which may be due to the direct effects of inflammation, poor nutrition, reduced lean-body mass, immobility^19^, and also to the effects of treatments for different medical conditions, such as T2D^42^. These mechanisms are quite complex and closely interrelated but are ultimately mediated through effects on the bone remodeling cycle. T2D is an inflammatory disease and is associated with an increased risk of osteoporotic fractures, resulting in disabilities and increased mortality^43^. Moreover, drugs used in the treatment of T2D, specifically PPARγ agonists, have also been reported to affect bone cell function and result in reduced BMD and increased risk of osteoporosis and fractures^17,44^. TZDs, a class of antidiabetic agents that act as insulin sensitizers, bind PPARγ in vivo with high affinity. PPARγ regulates target gene transcription as a heterodimer with the retinoid X receptor, which is synergistically activated by TZDs^45^. TZDs in general, and RSG in particular, enhance adipogenesis in stromal cells^46^. Activation of PPARγ stimulates adipogenesis and inhibits osteoblastogenesis in vitro in murine BM-derived clonal cell line^47^, and in vivo in mice; these processes are in turn associated with bone loss^48,49^. Both osteocytes and adipocytes are generated from the same MSCs progenitors. TZDs inhibit osteoblast differentiation and stimulate adipocyte differentiation from MSC as a potential mechanism through which they exert their bone detrimental effect^50,51^. Using different animal models of osteoporosis, we demonstrated that FO rich in omega-3 FAs attenuates osteoporotic bone loss by down-regulating inflammatory mediators^52–58^. In this study, we demonstrated that components of FO, co-administered for achieving a sinergistic effect and particularly DHA, can revert RSG-induced adipogenesis in a murine C3H10T1/2 cell line. Previously, it has been shown that RSG induces PPARγ expression and activity in primary BM stromal cells and C3H10T1/2 cells and that cyclic loading counteracts these effects^59^. In this study, we found a similar effect with FO supplementation in counteracting RSG-induced PPARγ expression in primary BM cells as well as RSG-induced adipogenesis in C3H10T1/2 cells, which prevented RSG-mediated bone loss in aging C57BL/6 mice. This is consistent with our earlier finding that feeding the FO diet in *fat1* transgenic mice prevented ovariectomy-induced bone loss by modulating the markers of inflammation and osteoclastogenic factors^60^.

Generally, ALP is considered to be an early marker of osteoblast differentiation. As ALP is a byproduct of osteoblast activity, an increased level of ALP refers to active bone formation^61^. It was previously shown that RSG-mediated ALP activity reduction in primary BM stromal cells and C3H10T1/2 cells can be counteracted by mechanical loading^59^. Similarly, we showed here that FO components, particularly DHA, can effectively counterbalance the known RSG deleterious action on BM stromal cells, C3H10T1/2 cells, and in an aging mouse model. Thus, FO supplementation prevents RSG-mediated activation of PPARγ leading to adipocyte differentiation and down-regulation of ALP activity as described for mechanical loading. This could be one of the mechanisms through which FO supplementation restores RSG treatment-associated bone loss in aging C57BL/6 mice. Besides, obesity and insulin resistance are characterized by both excess adipose tissue and osteoporosis^62^, two diverse conditions that are nonetheless linked at the point of stem cell differentiation. Adipocyte and osteoblast formation occurs from a common progenitor, MSC^63,64^, and signals that promote BM stromal cells differentiation toward one lineage may preclude the formation of the other. For example, when BM adiposity is increased in aging individuals, trabecular bone is essentially replaced by fat tissue^65^. Increased adipocyte formation also leads to the secretion of pro-inflammatory cytokines, such as IL-6 and TNF-α, which are associated with osteoclastogenesis and enhanced bone-resorbing activity. In our study, we found that RSG stimulated the formation of giant osteoclast cells in the RAW264.7 line when stimulated with RANKL, possibly by inducing the fusion of TRAP-positive mononuclear cells. Earlier, we demonstrated that omega-3 FAs, particularly DHA inhibits the fusion of TRAP-positive mononuclear cells in RAW264.7 cells when stimulated with RANKL^34^. Interestingly, the present study showed that omega-3 FAs treatment could attenuate RSG-mediated giant osteoclast formation in RAW264,7 cells. A significant reduction of TRAP activity of BM cells on dentin slice stimulated with PTHrp was also noted in RSG + FAs treated culture. This data reveals that FO supplementation may prevent bone loss secondary to RSG treatment by reducing osteoclastic bone resorption. One limitation in our cell culture studies is that we did not test for an independent effect of omega-3 FA on osteoblastogenesis, osteoclastogenesis, and adipogenesis in this current setup. Therefore, we could not determine exactly how much effect was achieved due to combining omega-3 FA and RSG. There is a conflicting report on the effect of RSG on osteoclast differentiation in RAW264.7 cells showing that RSG at a very high concentration can inhibit TNF-α induced osteoclastogenesis^66^. Compared to our study, the authors used a 20 times higher concentration of RSG and TNF-α, to stimulate osteoclast in RAW264.7 cells, instead of RANKL, which is the key stimulator of osteoclastogenesis in macrophage and bone marrow cells^67^. There are controversies on the role of TNF‐α in promoting osteoclastogenesis independently of RANKL^67^. Others demonstrated that RANKL priming in osteoclast precursors is necessary for TNF‐α‐induced osteoclastogenesis and TNF‐α is not necessarily required for osteoclastogenesis. We previously showed that RANKL treatment of macrophage cells induces the secretion of TNF‐α, which may stimulate the activation of pro-osteoclastogenic NF-κB and MAPK signaling^34^. Other groups also showed that RANKL induces TNF‐α mRNA expression and secretion of TNF‐α protein, which alone, in the absence of RANKL, could not induce osteoclastogenesis^68^. Therefore, TNF‐α might not be an appropriate stimulator to test the effect of a compound on osteoclastogenesis in the absence of RANKL.

Several studies demonstrated that omega-3 FAs alone or in combination with RSG improve insulin sensitivity^69–72^. Omega-3 FAs activate the anti-inflammatory GPR-120 pathway leading to enhance the systemic insulin sensitivity^70^. On the other hand, activation of GPR-120 is pro-bone-forming and anti-bone-resorbing^73,74^. These studies indicate that omega-3 FAs supplementation would not jeopardize RSG-mediated insulin sensitization; rather it might have further enhanced insulin sensitivity, in addition to its positive effects on bone metabolism. Importantly, increased adiposity in BM might not only suppress osteoblastogenesis but also promote osteoclastic bone resorption through the secretion of inflammatory cytokines by BM adipocytes, and other fat cells^75^. In this study, we found that the marker of bone resorption, cathepsin k^76^, and marker of adiposity, PPARγ^47^ mRNA levels are increased in LPS-stimulated BM cells collected from RSG-treated mice. However, FO co-administration restored both cathepsin k and PPARγ mRNA levels. This data further supports the notion that FO supplementation may prevent RSG-mediated bone destruction by reducing BM adiposity and osteoclastic bone resorption. GPR-120 activation might have been associated with FO co-administration-related reduction in adiposity and enhancement of BMD^73,74,77^. However, further studies are required to determine if the GPR-120 pathway plays a key role in FO co-administration-associated beneficial effect on bone.

It is well established that aging is linked with an increase in pro-inflammatory cytokines, such as IL-6 and TNF-α^78,79^. These cytokines are key regulators of osteoclastogenic activity and bone resorption^80,81^. Furthermore, they induce the expression of COX-II in osteoblastic and stromal cells, resulting in an increased production of PGE2, which is also an essential activator of osteoclastogenesis^82^. It is known that increased levels of COX-II stimulate osteoclastogenesis and bone resorption^41^. In this study, we found a lower activity of TNF-α, IL-6, and COX-II in BM cells isolated from RSG + FO treated mice, which could in part explain the maintenance of higher BMD compared with RSG alone treated mice. Previous studies have shown that omega-3 FAs decrease the expression and activity of these cytokines, both in vivo and in vitro^30,53^. We earlier demonstrated that immune cells and kidney tissues derived from FO-supplemented autoimmune disease-prone mice exhibited decreased levels of pro-inflammatory cytokines and lesser bone loss in these animals^53,83^. On the contrary, IL-10, an anti-inflammatory cytokine, has been shown to inhibit osteoclast formation and bone resorption in rat and mouse systems^84^. Interestingly, FO co-administration significantly increased the IL-10 levels.

In an effort to mimic in vitro the human setting in bone and adipose tissues during treatment with RSG and DHA, we also used the model of human MSCs, which are known to contribute to tissue repair and are capable of differentiating into osteoblasts and adipocytes^85^. This set of experiments confirmed the beneficial effect of DHA during treatment with RSG, through the down-regulation of adipogenic genes, such as adipsin and FABP4 along the PPARγ/FABP4 axis, which stands for a reduced capability of osteocytes to switch toward adipogenesis. On the other hand, there is a not concordant trend for osteogenic genes: RUNX2 expression is significantly increased while ALP is reduced in the cells treated with RSG and DHA compared to RSG alone.

Overall, the results on the adipogenic differentiation of MSCs are probably consistently biased by the low differentiation rate obtained on day 14, as per the manufacturer’s instructions. We believe that more consistent and reliable results on the effect of RSG and DHA on adipocytes could be obtained with an analysis conducted at a higher differentiation threshold, given that the changes in expression required for adipose differentiation, in terms of expression of a new set of genes and silencing of others, is significantly greater compared to the osteogenic differentiation^86^.

In addition to the already reported effect of FAs in the prevention of mitochondrial dysfuntion and oxidative stress associated to obesity and liver steatosis^87,88^ and as complementary therapies with RSG to counteract dyslipidemia and insulin resistance^69^, our study suggests that dietary FO supplementation helps to improve RSG-mediated bone loss in aging mice and human osteo-differentiated MSCs by modulating inflammatory and osteoclastogenic factors. Our study might find a direct translational clinical implementation of FAs in T2D patients on treatment with TZDs in attempt of preventing the known side effects of these drugs and reducing oxidative stress, pro-inflammatory state and liver steatosis in this category of patients^89,90^. Moreover, TZDs, as recognized potent weapons in clinical hands, might be extended to a larger scale and benefiting an increased number of patients in case a strategy to mitigate and/or prevent side effects is found and implemented.

Further experiments with combinations of RSG and FO in osteoporotic animal models, supplemented with purified enriched EPA or DHA diet (with and without treadmill exercise), and in vitro human models are necessary to clarify the mechanisms involved in the protection of bone loss, and also to determine their specific differential potential.

## Materials and methods

All media and cell culture components, such as Histopaque, lipopolysaccharide (LPS), 4′,6-diamidino-2-phenylindole, oil red O, l-ascorbic acid 2-phosphate, trypsin–EDTA reagent, BCIP/NBT buffered substrate tablets, RPMI-1640, α-Minimum Essential Medium (MEM), and PLTGold^®^ Human Platelet Lysate were purchased from Sigma-Aldrich Inc. (USA). Additional supplies were purchased from these respective sources: fatty acids (EPA and DHA): Cayman Chemical Company, Ann Arbor, MI; Murine soluble RANKL: Pepro Tech Inc. Rocky Hill, NJ; TRAP solution No. 387: Sigma Chemical Co., St. Louis, MO; BMP-2: Biovision, USA; RSG: Advance Scientific Inc. USA; penicillin and streptomycin: VWR; PTHrP: Bachem, USA; Qiashedder and RNeasy mini kits: Qiagen Inc. USA; One step cDNA synthesis kit for RT-PCR: Applied Biosystems, USA; GoTaq qPCR Master Mix: Promega, Madison, WI, USA; Bicinchoninic acid (BCA) protein assay kit: Pierce Inc, USA; Glucose (QuantiChrom, Hayward, CA), NEFA (Wako Pure Industries Ltd, Japan), ALP activity kit (ANASPEC, San Jose, CA) and insulin (Crystal Chem Inc. Research, Downers Grove, IL) were analyzed using commercially available kits following manufacturer’s instructions. Tumor Necrosis Factor-α (TNF-α), Interleukin 6 (IL-6), and Interleukin 10 (IL-10) were measured by the Ready-set-go ELISA kit (eBioscience, Inc. San Diego, CA) according to the manufacturer’s protocol. Differentiation kits for MSCs (StemPro™ Adipogenesis StemPro™ Osteogenesis Differentiation Kit) were bought from Gibco.

Samples from healthy volunteers are covered by Sidra Medicine IRB#1609004389. All animal studies were approved by the Institutional Animal Care and Use Committee (IACUC) of the University of Texas Health Science Center at San Antonio, US. All animal experiments were performed under the guidelines and regulations approved by IACUC.

### In-vitro experiments

#### Cell cultures

C3H10T1/2 and RAW 264.7 cells were grown in a T75-flask (BD Falcon, USA) in a 5% CO_2_ humidified atmosphere at 37 °C. The C3H10T1/2 cells (clone 8 American Type Culture Collection, USA) and RAW 264.7 were cultured in α-MEM, supplemented with 10% Fetal Bovine Serum (FBS) (HyClone Laboratories Inc. USA), l-glutamine, and 1% antibiotics (50 U/mL penicillin and 50 μg/mL streptomycin). After reaching a subconfluent state, C3H10T1/2 cells were trypsinized with 1 × trypsin–EDTA and plated into 48 or 96-well culture plates based on experimental need; the medium was then changed every other day.

#### Cell proliferation assay

C3H10T1/2 cells were plated on 96-well cell culture plates (2 × 10^4^ cell/well) in 100 μL of medium and grown for 24 h at 37 °C. Thereafter, RSG 1 µM was added with or without EPA 10 µM and/or DHA 10 µM and the cells were incubated for another 72 h. Cell proliferation was evaluated using an MTS proliferation assay kit (Promega Inc., USA)^57^.

#### Osteoblast differentiation in C3H10T1/2 cells

C3H10T1/2 cells were plated at a density of 2 × 10^3^ cells per 500 μL in a 48-well plate in α-MEM, supplemented with 10% FBS. After 24 h incubation, the media were replaced with osteogenic media (supplemented with BMP-2 100 ng/mL) in the presence of RSG 1 µM with or without EPA 10 µM and/or DHA 10 µM for 10 days. Media and factors were replaced every 3 days. Cells were then washed with cold phosphate buffer saline (PBS) and fixed with 10% formalin. Cells were washed 2 more times with PBS and incubated in 500μL of ALP solution (1 BCIP/NBT tablet to 10 mL distilled water) (Sigma Aldrich, St. Louis, MO) at room temperature (RT) for 10–15 min or until the desired staining was achieved. Wells were then carefully rinsed with PBS/EDTA 20 mM and the ALP-stained optical density (OD) intensity was analyzed by histomorphometry using the Metaview Image Analysis System (Olympus Inc. USA)^91^.

#### ALP activity in C3H10T1/2 cells

C3H10T1/2 cells were plated at a density of 2 × 10^3^ cells per 500 μL in a 48-well plate in α-MEM, supplemented with 10% FBS. After 24 h incubation, the media were replaced with osteogenic media (supplemented with BMP-2 100 ng/mL) in the presence of RSG 1 µM with or without EPA 10 µM and/or DHA 10 µM for 7 days. Media and factors were replaced every 3 days. Cells were then washed with cold PBS and incubated with 1× lysis buffer at 4 °C for 10 min with agitation. Cell suspensions were further centrifuged at 2500×*g* for 10 min at 4 °C. Supernatants were collected, and protein concentrations were quantified with BCA assays to assure uniform protein contents among all samples. ALP activity was measured by using SensoLyte pNPP ALP Assay (AnaSpec, San Jose, CA, USA) according to the manufacturer’s instructions. 50 μg of protein extracts were transferred in duplicate to a 96-well plate; the pNPP reaction mixture was added to each well, and plates were incubated for 30 min before reactions were stopped by adding the stop solution. ALP activity in each sample was determined by measuring OD405 and compared to an ALP standard curve.

#### Adipogenesis in C3H10T1/2 cells

Adipogenesis was measured by histochemical techniques, as described by Wang et al.^38^. In in-vitro experiments, C3H10T1/2 cells were plated in a 48-well plate and cultured in α-MEM medium with 10% FBS supplemented with rhBMP-2 (Biovision, USA) and cultured for 7 days in the presence of RSG 1 µM with or without EPA 10 µM and/or DHA 10 µM. Cells were then subjected to oil red O histochemical staining with fast red AL salt as a substrate. The OD of each well was measured using Metaview Image Analysis System (Olympus Inc. USA)^21^.

#### Osteoblast differentiation (Alizarin Red staining and ALP activity) in BM stromal cells

Stromal cells were generated from whole BM isolated from the femurs and tibias of 4–6 week old C57BL/6 female mice obtained from Jackson Laboratories (Bar Harbor, Maine). Cells were plated in α-MEM, supplemented with 10% FBS and 1% penicillin–streptomycin (Sigma Aldrich, St. Louis, MO) in a T75 culture flask, and incubated at 37 °C at 5% CO_2_. After 24 h of incubation, non-adherent cells were removed by replacing them with a fresh medium. Cells were allowed to grow to confluency and split at 1:2 ratios for a minimum of 6 weeks. The remaining viable cells generally belong to BM stromal type lineage. BM stromal cells were then plated at a density of 2 × 10^4^ cells per 100 μL in a 96-well plate in α-MEM, supplemented with 10% FBS. After 24 h of incubation, media were replaced with osteogenic media (supplemented with Ascorbic Acid 50 μM and β-Glycerophosphate (Sigma Aldrich, St. Louis, MO) 10 mM in the presence of RSG 1 µM with or without EPA 10 µM and/or DHA 10 µM and incubated for an additional 7–10 days with media, factors and fatty acid changes every 2 days. Osteoblast differentiation was determined by two sets of experiments: Alizarin Red staining and ALP activity. For Alizarin Red staining, cells were washed with PBS and fixed with 10% formalin by incubating the plates at room RT for 15 min at the end of 10 days culture. Cells were then washed 3 times with PBS, avoiding disturbing the fragile monolayer of cells. After the final aspiration, Alizarin Red S Solution 50μL (Alizarin Red S 2 g, distilled water 100 mL, adjusted pH 4.1 to 4.3 using 0.5% ammonium hydroxide) (Sigma Aldrich, St. Louis, MO) was added for 20 min at RT. Wells were then carefully washed with distilled water and Alizarin Red S stained OD intensity was determined by histomorphometry using the Metaview Image Analysis System software^91^. For ALP activity, at the end of 7 days culture, cells were washed with cold PBS and incubated with 1× lysis buffer at 4 °C for 10 min with agitation. Cell suspensions were further centrifuged at 2500×*g* for 10 min at 4 °C. Supernatants were collected, and protein concentrations were quantified with BCA assays to assure uniform protein contents among all samples. ALP activity was measured by using SensoLyte pNPP ALP Assay (AnaSpec, San Jose, CA, USA) according to the manufacturer’s instructions. 50y μg of protein extracts were transferred in duplicate to a 96-well plate; the pNPP reaction mixture was added to each well, and plates were incubated for 30 min before reactions were stopped by adding the stop solution. ALP activity in each sample was determined by measuring OD405 and compared to an ALP standard curve.

#### Osteoclast differentiation in RAW264.7 cells

RAW264.7 cells were suspended in α-MEM containing FBS 10% and plated at a concentration of 2 × 10^4^ cells/well into a 48-well tissue culture plate in the presence of RANKL 50 ng/mL with or without RSG 1 µM, with or without EPA 10 µM and/or DHA 10 µM. The medium, factors, and drugs were replaced after 3 days. After 5 days of culture, the cells were fixed and stained for TRAP using a TRAP staining kit according to the manufacturer’s instruction. TRAP-positive cells with more than three nuclei were counted as TRAP-positive multinucleated cells (MNCs) and as osteoclast-like cells^34^.

#### TRAP activity of mouse BM cells on dentin slice

Sperm whale dentin slices were prepared as described earlier^92^ using a Buehler low-speed diamond bone saw (Buehler, Lake Bluff, IL, USA) followed by sonication (15 min) in several changes of distilled water. Slices were polished between two glass plates, transferred to Petri dishes, and sterilized under UV light for 1 day. Slices were soaked in 100% ethanol, allowed to air dry, and rinsed twice with sterile saline solution. Before all experiments, slices were incubated in α-MEM supplemented with FBS 10% and penicillin–streptomycin solution 1% for at least 24 h before use.

Four-week-old C57Bl/6 female mice were purchased from Jackson Laboratories (Bar Harbor, Maine 04609 USA). Whole BM cells were collected aseptically from the femora and tibiae, and plated in α-MEM, supplemented with FBS 10% and penicillin–streptomycin solution 1% in a T75 culture flask, and incubated at 37 °C at 5% CO_2_. After 4 h of culture, non-adherent cells (osteoclast precursor cells) were collected, 1 × 10^6^ cells/500 µL/well were gently plated on the dentine slices placed on a 48-well tissue plate and cultured in the presence or absence of 40 ng/mL synthetic human PTHrP for 7 days at 37 °C in a humidified 95:5 atmosphere of air/CO_2_ (mol%) with or without RSG 1 µM, with or without EPA 10 µM and/or DHA 10 µM as described earlier^93^. The medium, factors, and drugs were replaced once in 3 days. A minimum of 3–4 slices was used per treatment.

For TRAP activity measurement on dentin slices, the medium was removed at the end of the culture, and then the cell monolayer was gently washed twice with PBS. The cells were then lysed with 200 mL of 0.2% Triton X-100. TRAP activity in cell lysate was determined using the TRAP solution No. 387 kit. An aliquot of cell lysate was added to 150 mL TRAP solution and was incubated at 37 °C for 30 min. The absorbance was then measured at 570 nm using a microplate reader^34^.

### In-vivo experiments

#### Mice strain

Ten-month-old female C57Bl/6 mice, weighing 23–25 g, were purchased from Jackson Laboratories (Bar Harbor, Maine 04609 USA) and fed a standard diet (Harlan Teklad LM-485) for 3 months. Weight-matched animals were then divided into five groups (n = 10 per group) and housed in a standard controlled animal care facility. The animals were maintained in a temperature-controlled room (22–25 °C, 45% humidity) on a 12:12 h dark–light cycle. The mice were fed an American Institute of Nutrition diet (AIN93) supplemented with fat, with 10% of total calories coming from either CO or CO plus FO, in a 50/30/20 ratio of CO, EPA, and DHA, respectively. RSG was administered to half animals in each diet group (RSG + CO and RSG + CO + FO). All animals were fed ad libitum for 5 additional months. A fifth experimental group fed a standard rodent LC diet, was also maintained throughout the experiment. Body weights and food intake of mice were measured weekly.

#### FO dietary supplement regime with RSG in C57Bl/6 aging mice

Five different dietary regimes were as follow: I) 10%CO; II) 5%CO and 5%FO; III) 10%CO plus RSG (RSG + CO); IV) 5%FO + 5%CO plus RSG (RSG + FO) and V) standard rodent LC pelleted diet. The composition of the semi-purified AIN93 diet is presented in Table S1. Groups I–IV were fed their experimental diets, produced by an in-house diet preparation facility, for 5 months. RSG-treated animals (groups III and IV) were fed 5 g of food per day, supplemented with RSG maleate at a concentration of 0.14 mg/g. Non-RSG-treated mice (groups I, II, and V) were fed the same quantity (5 g/day) of CO diet, FO diet, or LC, respectively, similar to those used previously^94^. The mice were provided with fresh food every day in the afternoon (between 1:00 and 2:00 pm). The remaining food was removed daily to prevent rancidity. Diets were prepared each week, purged with nitrogen gas, frozen in daily portions, and sealed in polyethylene bags to minimize the oxidation of the fatty acids.

#### BMD measurements

BMD was determined using the Piximus instrument and software version 2.1 (GE Lunar, Madison, WI). Mice were anesthetized and scanned at 13 months of age, before the onset of the experimental diet, and then before sacrifice, at age 18 months. Total body BMD (g/cm^2^), excluding the head region, was obtained from each scan, and the percent change in BMD was determined as previously described^30^.

#### Isolation of whole BM cells from aging C57BL/6 mice with experimental diets and determination of bone markers

After 5 months of experimental diets, whole BM cells were aseptically isolated at the time of sacrifice as described previously^53^. Cells were counted and viability was determined by the trypan blue exclusion method. Cells (10 × 10^6^/well) were plated in 6-well plates and treated with bacterial LPS at a concentration of 5.0 μg/mL for 24 h at 37 °C, in a humidified atmosphere of air/CO_2_ in a ratio of 95:5 (mol%). After 24 h, cells and culture medium were collected together and centrifuged at 2000 rpm for 5 min. The cell pellets and culture supernatants were stored separately at − 80 °C. Cell pellets were used for gene expression, ALP (AnaSpec kit), and COX-II activity assays, while culture supernatants were used to analyze the levels of TNF-α, IL-6, and IL-10 cytokines.

#### Splenocyte preparation and culture

Spleens were aseptically removed from diet-fed mice at the time of sacrifice and placed in 5 mL of RPMI 1640 medium supplemented with HEPES 25 mmol/L, glutamine 2 mmol/L, penicillin 100,000 U/L, and streptomycin 100 mg/L. Single-cell suspensions were made by teasing spleens between frosted ends of two sterile glass slides. After 5-min centrifugation at 100×*g* to separate cells from debris, the cells were washed twice in RPMI medium. Splenic lymphocytes were isolated by layering over Histopaque, centrifuging at 1000 rpm for 15 min at 22 °C, and then washing twice in RPMI 1640 complete medium. Cells were plated in 6-well plates (10 × 10^6^ cells/well) and viability was determined by the trypan blue exclusion method; bacterial LPS was added at a concentration of 5.0 µg/mL for 24 h at 37 °C in a humidified 95:5 atmosphere of air/CO_2_ (mol%). After 24 h, the culture medium was collected and analyzed for TNF-α, IL-6, and IL-10 by standard ELISA techniques^55^.

#### Cytokines measurement in the conditioned medium of cultured splenocytes and BM cells

TNF-α, IL-6, and IL-10 were measured in conditioned media by Ready-set-go ELISA kit (eBioscience, Inc. San Diego, CA) according to manufacturer’s protocol^55^.

#### RNA extraction and real-time RT-PCR

Real-time RT-PCR was used to determine the mRNA levels of cathepsin k and PPARγ. RNA was extracted from LPS-stimulated BM cells using the RNeasy Mini Kit (Qiagen, Valencia, CA) according to the manufacturer’s instructions. The concentration of RNA was determined using NanoDrop™ (Thermo Scientific, Wilmington, DE, USA). Real-time RT-PCR was then carried out using TaqMan^®^ RNA-to-C_T_ 1-step kit (Applied Biosystems, Foster City, CA) in an ABI Prism 7900HT Sequence Detection System (Applied Biosystems) using fluorescent TaqMan system. Real-time quantitative RT-PCR was performed for each of the following genes using ready-to-use primer and probe sets developed by Applied Biosystems (TaqMan Gene Expression Assays): cathepsin k (*Ctsk*, Mm00484036_m1), PPARγ (*pparγ*, Mm01184321_m1) and Glyceraldehyde-3-phosphate dehydrogenase (*Gapdh*, Mm99999915_g1) as an endogenous control. mRNA Ct values for these genes were normalized to the house-keeping gene GAPDH and expressed as relative increase or decrease, compared with the CO group^95,96^.

#### ALP quantification in BM cells

ALP activity was measured by using SensoLyte pNPP ALP Assay kit in 50 μg protein extracts of 24 h-cultured BM cells from experimental-diet and LC-fed mice, as described in in-vitro experiments.

#### Cyclo-oxygenase-II (COX-II) activation assay

100 μg of cytosolic protein of 24 h-LPS-treated BM cells from aging C57BL/6 mice with experimental diets were analyzed for COX-II activity using CAYMAN COX Activity Assay Kit according to the manufacturer’s instruction^55^.

### Human readout in vitro

#### AD-MSC culture and differentiation

Human MSCs were isolated from discarded lipoaspirate of 2 healthy volunteers (Sidra Medicine IRB#1609004389). Automated and clinical-grade fat digestion was performed through the Celution^®^ 800/CRS System (Cytori Therapeutics Inc., San Diego, USA). The isolated cells were seeded and cultured in flasks for expansion (2 × 10^6^ cells in T75 flask) with 5% PLTGold^®^ Human Platelet Lysate (Sigma-Aldrich), low-glucose Dulbecco’s Modified Eagle Medium (DMEM) (Sigma-Aldrich), 1% 10,000 U/mL penicillin, and 10 mg/mL streptomycin (VWR), at 37 °C in a humidified 95:5 atmosphere of air/CO_2_ (mol%). The AD-MSCs were adherent to plastic, displayed fibroblastoid morphology, and were compliant with the International Society of Cell Therapy criteria for MSCs^97^. AD-MSCs were then cultured in adipo- (StemPro™ Adipogenesis Differentiation Kit (Gibco)) and osteo- (StemPro™ Osteogenesis Differentiation Kit (Gibco)) differentiating media, for 14 and 21 days respectively, as per manufacturer’s instructions, with the following conditions: AD-MSCs (control), AD-MSCs + RSG 1 µM (Cayman Chemical Company), AD-MSCs + DHA 10 µM (Cayman Chemical Company), AD-MSCs + RSG + DHA. Each condition was run in duplicate for RNA extraction and staining.

#### AD-MSCs staining

AD-MSCs were fixed with 4% Paraformaldehyde for 30 min and washed with water. Adipo-cultures were treated with 60% isopropanol for 5 min, stained with Oil Red O (Sigma) for 45 min, washed, and dried at RT. Osteo-cultures were directly stained with Alizarin-Red Staining Solution (Merck Millipore) for 45 min, gently washed, and finally dried at RT.

The plates were observed and photographed at an inverted microscope at 4×, 10×, and 20× and images were analyzed with the ImageJ software.

#### RNA isolation and quantitative real-time RT-PCR

Total RNA was extracted from differentiated AD-MSCs using Trizol reagent (Invitrogen) according to the manufacturer’s instructions. The isolated RNA was DNase treated followed by first-strand cDNA using SuperScript™ III First-Strand Synthesis SuperMix (Invitrogen). Semi-quantitative analysis of relative gene expressions was performed on the QuantStudio™ 12 K Flex System (Applied Biosystems, Carlsbad, CA) using GoTaq qPCR Master Mix (Promega, Madison, WI, USA). Primers were synthesized by Integrated DNA Technologies (Coralville, CA), sequences are listed in Table S2. All primer pairs were validated by demonstrating high amplification efficiency and a consistent single peak melt curve. The relative gene expression was normalized to β-Actin (endogenous control) and calculated using the 2^−ΔΔCt^ method setting the values of control as one.

### Statistical analysis

Results are expressed as mean ± SE. Data were statistically analyzed using one-way ANOVA; p < 0.05 was considered statistically significant. Newman–Keuls and Dunnett’s multiple-comparison tests were used to test the significance of differences among group means.

## Supplementary Information

Supplementary Information.

## Data Availability

All data generated or analyzed during this study are included in this published article and its supplementary information file.
