# Supplementary material for: Omega-3 fatty acid-rich fish oil supplementation prevents rosiglitazone-induced osteopenia in aging C57BL/6 mice and in vitro studies
Source: Sci Rep. 2021 May 14;11:10364. doi: 10.1038/s41598-021-89827-8 (PMC8121944; doi:10.1038/s41598-021-89827-8)
Supplement: Supplementary file 1 — Supplementary Information. [file 41598_2021_89827_MOESM1_ESM.pdf]

OMEGA-3 FATTY ACID-RICH FISH OIL SUPPLEMENTATION PREVENTS ROSIGLITAZONE-INDUCED OSTEOPENIA IN AGING C57BL/6 MICE AND *IN VITRO* STUDIES.

Authors' names and Institution: Chiara Cugno[1], Dhanya Kizhakayil[1], Rita Calzone[1], Shaikh Mizanoor Rahman[2], Ganesh V Halade[3] and Md M Rahman[4]*

Affiliations:

[1] Advanced Cell Therapy Core, Sidra Medicine, Doha, Qatar.

[2] Natural & Medical Sciences Research Center, University of Nizwa, Sultanate of Oman.

[3] Division of Cardiovascular Sciences, the University of South Florida Health, Tampa, Florida.

[4] Department of Biological and Environmental Sciences, Qatar University, Doha, Qatar.

***Corresponding author**

Md Mizanur Rahman, PhD Email: mrahman@qu.edu.qa

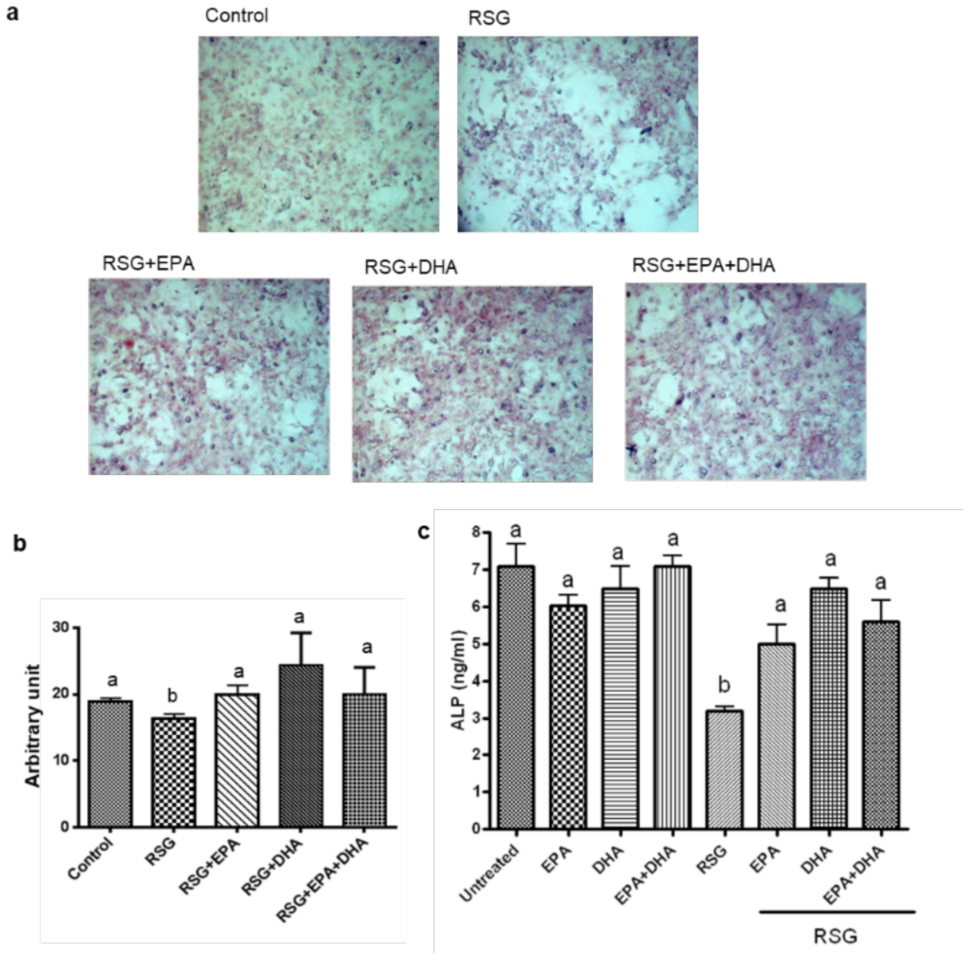

Supplemental Figure 1. Effect of RSG with or without omega-3 FAs on osteogenesis in bone marrow stromal cells

Bone marrow cells were isolated from C57BL/6 mice and cultured in α-MEM supplemented with 10% FBS to generate bone marrow (BM) stromal cells. BM stromal cells were used to visualize matrix calcification by alizarin red staining and osteoblast differentiation by alkaline phosphatase activity. To determine the effect on the matrix calcification, BM stromal cells were cultured in the presence of ascorbic acid and β-glycerophosphate with RSG 1 μM with or without EPA 10 μM and/or DHA 10 μM for 10 days. Cells were then fixed and stained for Alizarin Red. **a)** Representative photographs (20x) of BM stromal cells stained for alizarin red S. **b**) Optical Density (OD) intensity was determined by histomorphometry using the Metaview Image Analysis System software for Alizarin Red positive area. **c**)To determine the effect on osteoblast differentiation, BM stromal cells were cultured in the presence of ascorbic acid and β-glycerophosphate with RSG 1μM with or without EPA 10μM and/or DHA 10μM for 7 days. Then cell lysates were prepared and ALP activity was measured in cell lysates by using SensoLyte pNPP ALP Assay (AnaSpec, San Jose, CA, USA). ALP activity in each sample was determined by measuring OD405 and compared to an ALP standard curve. Each bar represents the mean ± SEM of two independent quadruplicate cultures. Value with different superscripts is significantly different at $P<0.05$ by Newman Keuls one way ANOVA with multiple comparison test.

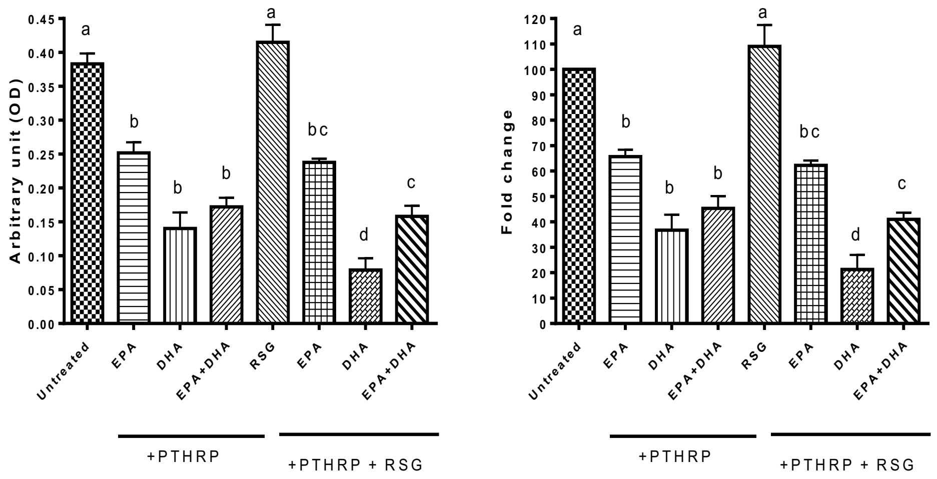

Supplemental Figure 2. Effect of RSG with or without omega-3 FAs on osteoclastic activity in bone marrow cells.

Bone marrow (BM) cells isolated from C57BL/6 mice were cultured in α-MEM containing 10% FBS with 40 ng/mL PTHrP in the presence of RSG 1μM with or without EPA 10μM and/or DHA 10 μM for 7 days on sperm whale dentin slice placed in a 48 well tissue culture plate. The cells were then lysed with 200 mL of 0.2% Triton X-100. Tartrate-resistant acid phosphatase (TRAP) activity in cell lysate was determined using a TRAP solution. The absorbance was then measured at 570 nm using a microplate reader. Each bar represents the mean ± SEM of two independent quadruplicate cultures. Value with different superscripts is significantly different at $P<0.05$ by Newman Keuls one way ANOVA with multiple comparison test.

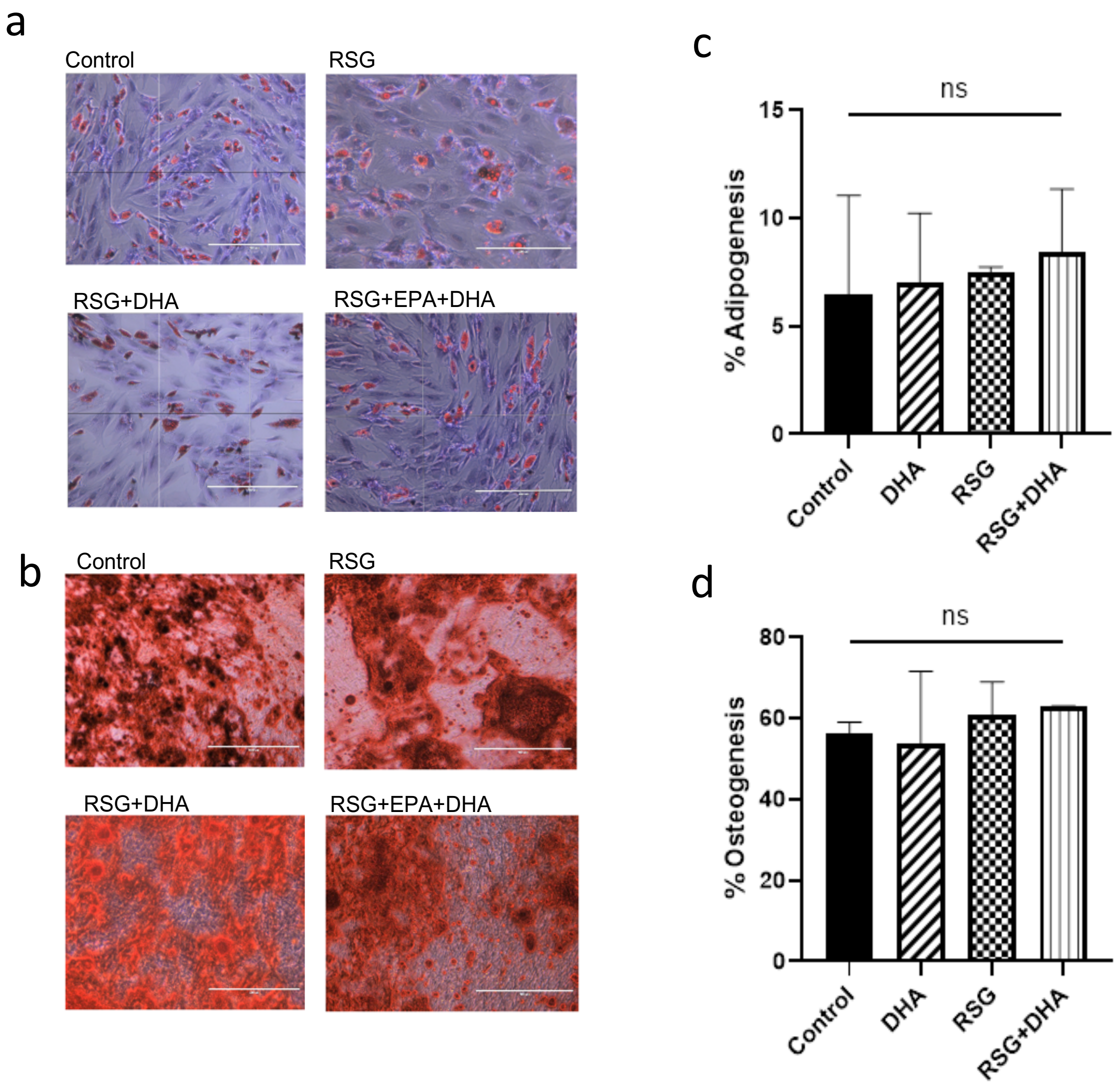

Supplemental Figure 3. Effect of RSG with or without FO on adipo- and osteo-differentiation of human Mesenchymal Stromal Cells (MSCs). Adipose-derived-MSCs were differentiated in an adipogenic medium (a) and in an osteogenic medium (b) with the presence or absence of RSG, DHA, and combination of RSG and DHA. The plates were photographed at an inverted microscope at 4x, 10x, and 20x and images were analyzed with the ImageJ software. The percentage of adipo- and osteo-differentiated MSC at the different conditions was calculated (c,d) [ns- P-value non-significant] n=2 donors. RSG, rosiglitazone; EPA, Eicosapentaenoic acid; DHA, Docosahexaenoic acid.

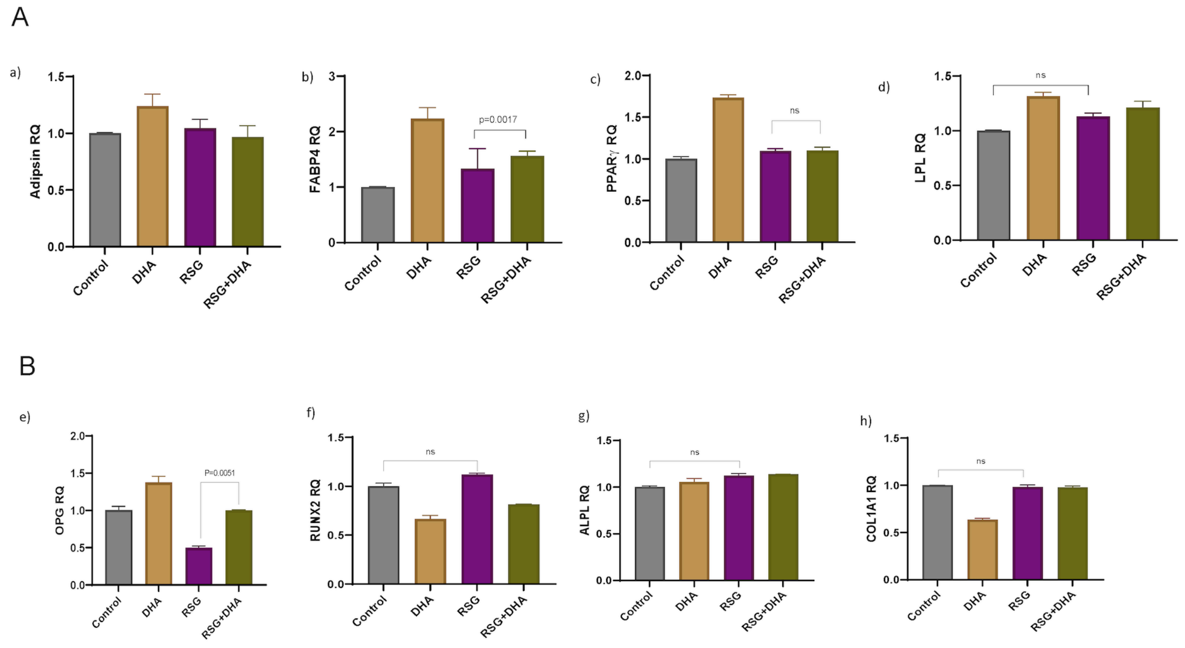

Supplemental Figure 4. Effect of RSG with or without FO on gene-expression of adipo-differentiated Mesenchymal Stromal Cells (MSCs).

Gene expression of adipogenic (**a**) and osteogenic (**b**) markers in MSCs differentiated in adipogenic medium with the presence of RSG, DHA and combination of RSG and DHA. Relative quantification of gene expression i) Adipsin, ii) FABP4, iii) LPL, iv) PPARγ, v) OPG, vi) RUNX2, vii) ALPL, viii) COL1A1. One-way ANOVA test followed by Dunnett's multiple comparisons test, P values are marked against RSG; ns- P value non-significant. n = 2 donors.

Supplemental Table 1. Composition of semi-purified experimental diets

**Ingredients**[a]	**CO**	**FO**	**CO+RSG**	**FO+RSG**
Casein	14.00	14.00	14.00	14.00
Corn starch	42.43	42.43	42.43	42.43
Dextronized corn starch	14.50	14.50	14.50	14.50
Sucrose	9.00	9.00	9.00	9.00
Cellulose	5.00	5.00	5.00	5.00
AIN-93 mineral mix	3.50	3.50	3.50	3.50
AIN-93 vitamin mix	1.00	1.00	1.00	1.00
l-cystine	0.18	0.18	0.18	0.18
Choline bitartrate	0.25	0.25	0.25	0.25
TBHQ	0.10	0.10	0.10	0.10
Vitamin E	0.04	0.04	0.04	0.04
rosiglitazone maleate	0.00	0.00	0.014	0.014
CO	10.00	0.00	10.00	0.00
FO	1.00	9.00	1.00	9.00

[a] All diet ingredients (expressed as percent total diet) from MP Biomedicals (Irvine, CA) and FO (EPA/DHA, 30/20) from Ocean Canada Ltd.

Supplemental Table 2. Panel of tested genes and PCR primers used for gene expression studies on differentiated AD-MSCs.

**Gene**	**Sequence (Sense and antisense 5'-3')**	**Amplicon size (bp)**	**Marker**
ADPS, Adipsin	TGAAGGTCAGGGTCACCCAA	72	Adipogenesis
	AAGACCAACCAGATGCAGGAG		
FABP4, fatty acid binding protein 4	GCCAGGAATTTGACGAAGTCAC	88	Adipogenesis
	TTCTGCACATGTACCAGGACAC		
LEP, Leptin	TCTTGTGGCTTTGGCCCTATCT	181	Adipogenesis
	CCAGTGTCTGGTCCATCTTGGATA		
PPARγ 2, peroxisome proliferator activated receptor γ	CTATGGAGTTCATGCTTGTG	134	Adipogenesis
	GTAC TGAGTACTGACA TTTATTT		
LPL, lipoprotein lipase	TGTAGATTCGCCCAGTTTCAGC	490	Adipogenesis
	AAGTCAGAGCCAAAAGAAGCAGC		
RUNX2, RUNX family transcription factor 2	GCCTTCAAGGTGGTAGCCC	182	Early osteogenesis
	AAGGTGAAACTCTTGCCTCGTC		
OPG, osteoprotegerin	GCTAACCTCACCTTCGAG	335	Early osteogenesis
	TGATTGGACCTGGTTACC		
ALPL, Alkaline phosphates	AGCTGAACAGGAACAACGTGA	135	Early osteogenesis
	CTTCATGGTGCCCGTGGTC		
COL1A1, collagen type I α-chain	TCTGCGACAACGGCAAGGTG	146	Early osteogenesis
	GACGCCGGTGGTTTCTTGGT		
BGLAP, bone γ-carboxyglutamate protein	CGCCTGGGTCTCTTCACTAC	141	Late osteogenesis
	CTCACACTCCTCGCCCTATT		
OPN, osteopontin	TTGCAGCCTTCTCAGCCAA	76	Late osteogenesis
	GGAGGCAAAAGCAAATCACTG		
β-Actin	AGAGCTACGAGCTGCCTGAC	111	Housekeeping gene
	GGATGCCACAGGACTCCA		
